# Full-length transcriptome sequences by a combination of sequencing platforms applied to isoflavonoid and triterpenoid saponin biosynthesis of *Astragalus mongholicus* Bunge

**DOI:** 10.1186/s13007-021-00762-1

**Published:** 2021-06-15

**Authors:** Minzhen Yin, Shanshan Chu, Tingyu Shan, Liangping Zha, Huasheng Peng

**Affiliations:** 1 School of Pharmacy, Anhui University of Chinese Medicine, Hefei, 230012 China; 2 State Key Laboratory of Dao-Di Herbs, National Resource Center for Chinese Materia Medica, China Academy of Chinese Medical Sciences, Beijing, 100700 China; 3 Research Unit of DAO-DI Herbs, Chinese Academy of Medical Sciences, 2019RU57, Beijing, 100700 China; 4 Anhui Province Key Laboratory of Research & Development of Chinese Medicine, Hefei, 230012 China; 5 Institute of Conservation and Development of Traditional Chinese Medicine Resources, Anhui Academy of Chinese Medicine, Hefei, 230012 China

**Keywords:** *Astragalus mongholicus* Bunge, Isoflavonoid, Triterpenoid saponin, Transcriptome, Biosynthesis

## Abstract

**Background:**

*Astragalus mongholicus* Bunge is an important medicinal plant used in traditional Chinese medicine. It is rich in isoflavonoids and triterpenoid saponins. Although these active constituents of *A. mongholicus* have been discovered for a long time, the genetic basis of isoflavonoid and triterpenoid saponin biosynthesis in this plant is virtually unknown because of the lack of a reference genome. Here, we used a combination of next-generation sequencing (NGS) and single-molecule real-time (SMRT) sequencing to identify genes involved in the biosynthetic pathway of secondary metabolites in *A. mongholicus*.

**Results:**

In this study, NGS, SMRT sequencing, and targeted compound analysis were combined to investigate the association between isoflavonoid and triterpenoid saponin content, and specific gene expression in the root, stem, and leaves of *A. mongholicus*. Overall, 643,812 CCS reads were generated, yielding 121,107 non-redundant transcript isoforms with an N50 value of 2124 bp. Based on these highly accurate transcripts, 104,756 (86.50%) transcripts were successfully annotated by any of the seven databases (NR, NT, Swissprot, KEGG, KOG, Pfam and GO). Levels of four isoflavonoids and four astragalosides (triterpenoid saponins) were determined. Forty-four differentially expressed genes (DEGs) involved in isoflavonoid biosynthesis and 44 DEGs from 16 gene families that encode enzymes involved in triterpenoid saponin biosynthesis were identified. Transcription factors (TFs) associated with isoflavonoid and triterpenoid saponin biosynthesis, including 72 MYBs, 53 bHLHs, 64 AP2-EREBPs, and 11 bZIPs, were also identified. The above transcripts showed different expression trends in different plant organs.

**Conclusions:**

This study provides important genetic information on the *A. mongholicus* genes that are essential for isoflavonoid and triterpenoid saponin biosynthesis, and provides a basis for developing the medicinal value of this plant.

**Supplementary Information:**

The online version contains supplementary material available at 10.1186/s13007-021-00762-1.

## Background

The genus *Astragalus mongholicus* Bunge, family Fabaceae (Leguminosae), contains perennial herbaceous plants, many of which are conventional therapeutic plants with a long history in traditional popular medicine in China [1, 2]. *A. mongholicus* has been broadly utilized as a principal herbal medicine in China, including Northeast, North, and Northwest China, as well as in Mongolia and Korea [3]. Based on pharmacological studies and clinical practice, the dried roots of *A. mongholicus* have good potential therapeutic applications, including vital-energy tonification, abscess drainage, diuretic properties, skin reinforcement, and tissue regeneration [4–6]. Recent studies revealed that these bioactivities are mainly associated with isoflavonoids and triterpenoid saponins [7–9]. Isoflavonoids and triterpenoid saponins are widely distributed in plants, and their biosynthetic pathways are well understood, especially in some model plants [10, 11]. However, *A. mongholicus* is not a model organism and little genomic information is available for this plant. This profoundly limits the research into the regulatory mechanisms of development, metabolite biosynthesis, and other physiological processes in this plant.

The isoflavonoid biosynthetic pathway is a branch of the phenylpropanoid pathway [12, 13]. Isoflavonoids are a class of phenolic secondary metabolites that regulate the complex plant–microbe interactions and are mainly synthesized by legumes [14, 15]. Phenylalanine ammonia-lyase (PAL) is the first key enzyme in the phenylpropanoid pathway; cinnamate 4-hydroxylase (C4H) is the second enzyme in the pathway; and 4-coumarate CoA-ligase (4CL) produces *p*-coumaroyl-CoA as a precursor for the synthesis of chalcone. Chalcone synthase (CHS) catalyzes the conversion of *p*-coumaroyl-CoA to naringenin chalcone in the first committed step of flavonoid biosynthesis. Next, chalcone isomerase (CHI) catalyzes the cyclization of the chalcones, producing liquiritigenin or naringenin, which act as substrates for many downstream enzymatic reactions for ultimate flavonoid formation in leguminous plants. As the key step at the very beginning of the isoflavonoid metabolic pathway, isoflavone synthase (IFS) converts flavanones to their corresponding isoflavonoids.

Current research on the biosynthesis of triterpenoid saponins can be divided into three stages: synthesis of upstream precursors, synthesis of the intermediate carbocyclic skeleton, and downstream formation of different types of triterpenoid saponins by various complex functionalization reactions [16]. The upstream precursors isopentenyl diphosphate (IPP) and dimethylallyl diphosphate (DMAPP) are mainly formed via the mevalonic acid pathway (MVA) and the methylerythritol pathway (MEP) [17–19]. The carbocyclic skeleton of triterpenoid saponins is synthesized from DMAPP in reactions catalyzed by prenyltransferase and oxidosqualene cyclases (OSCs) [20, 21]. In addition, different types of triterpenoid saponin products are generated by chemically modified cytochrome P450 monooxygenases (CYP450s), UDP-glycosyltransferases (UGTs), and glycosidases.

Transcriptome studies provide a useful perspective on the molecular biology of medicinal plants, including molecular mechanisms of gene function, cellular response, and different biological processes [22–26]. In the current study, we combined next-generation sequencing (NGS) and single-molecule real-time (SMRT) sequencing to obtain a complete *A. mongholicus* transcriptome, as a valuable resource for studying the regulatory mechanisms of development, metabolite biosynthesis, and other physiological processes of *A. mongholicus* in the absence of the available reference genome. We then used ultra-high performance liquid chromatography-tandem mass spectrometry (UPLC-MS/MS) to determine the levels of four primary isoflavonoids and four main astragalosides (triterpenoid saponins) in the root, stem, and leaves of this plant, to investigate the isoflavonoid and triterpenoid saponin biosynthesis in *A. mongholicus*. Candidate key genes involved in isoflavonoid and triterpenoid saponin biosynthesis in *A. mongholicus* were then identified. This study provides novel insights into the isoflavonoid and triterpenoid saponin biosynthesis in *A. mongholicus*.

## Results

### Accumulation of isoflavonoids and triterpenoid saponins in different organs of *A. mongholicus*

**Fig. 1:**
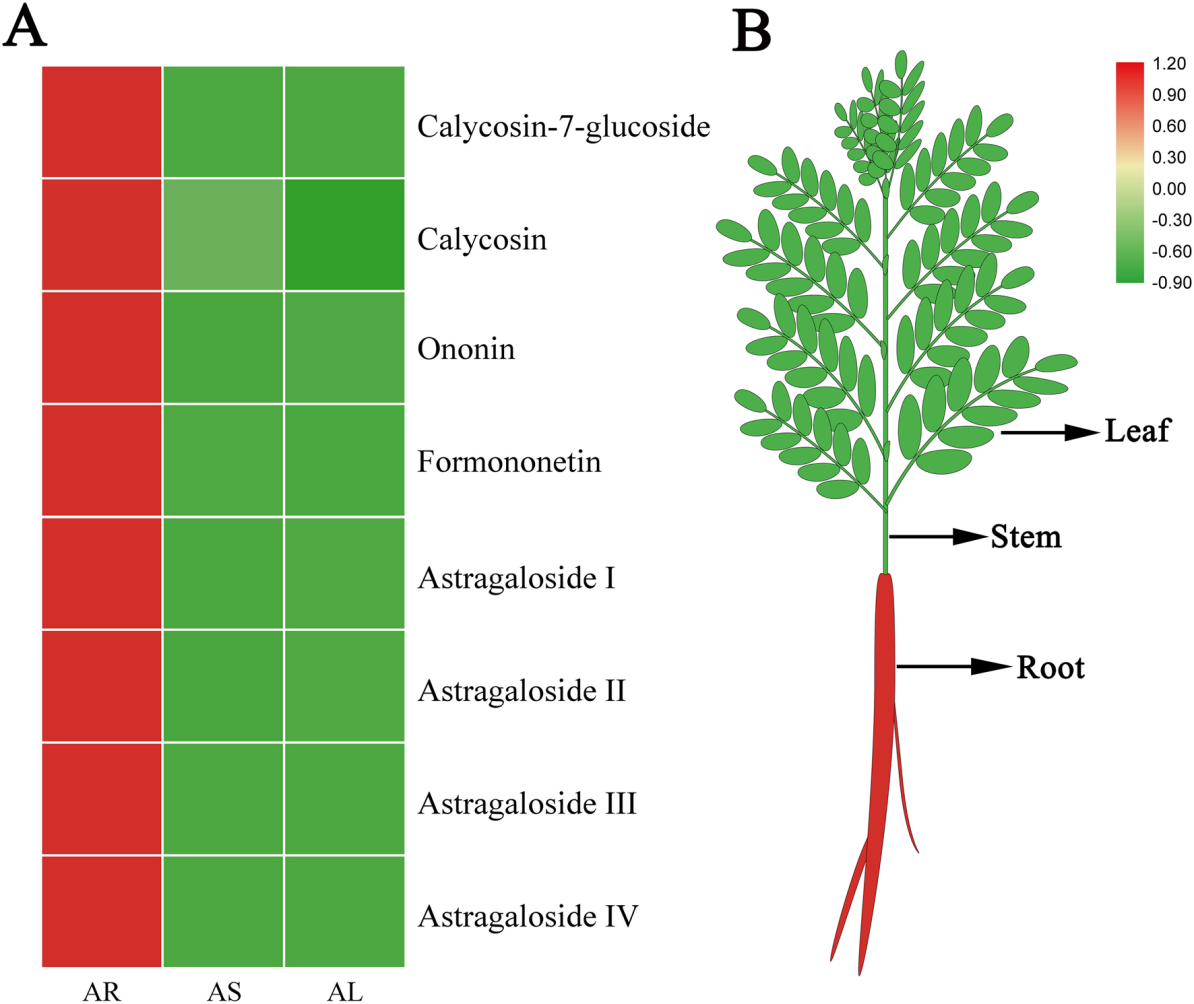
Distribution and accumulation of four isoflavonoids and four triterpenoid saponins in root, stem, and leaves of *A. mongholicus*. **a** Heatmap of four isoflavonoids and four triterpenoid saponins. **b** The eight components were mainly accumulated underground. *AR* root, *AS* stem, *AL* leaves

Four isoflavonoids and four triterpenoid saponins were identified by UPLC-MS/MS in the root (AR), stem (AS), and leaves (AL) of *A. mongholicus*, based on comparison of their retention times and MS fragmentation patterns with those of standard compounds. As shown in Fig. 1, calycosin-7-glucoside (0.244 ± 0.085 mg/g, DW), calycosin (0.086 ± 0.036 mg/g, DW), ononin (0.0483 ± 0.026 mg/g, DW), and formononetin (0.008 ± 0.005 mg/g, DW) were abundant in AR. For triterpenoid saponins, astragaloside I (0.993 ± 0.447 mg/g, DW), astragaloside II (0.078 ± 0.023 mg/g, DW), astragaloside III (0.018 ± 0.007 mg/g, DW), and astragaloside IV (0.146 ± 0.137 mg/g, DW) were also abundant in AR.

### Transcriptome analysis of *A. mongholicus* using a combined sequencing approach

To obtain as many high-quality unigenes as possible, and differentiate the stems, leaves transcriptomes, a hybrid sequencing strategy that combined SMRT and NGS technology was used. First, nine mRNA samples from three different organs (the root, stem, and leaves) were sequenced using the DNBSEQ platform. The raw data contained low-quality reads, sequencing adapters, and unknown bases (“N” bases). These reads were removed before data analysis to ensure the reliability of the results. After quality filtering, 132.46, 133.18, and 129.68 M of clean reads were obtained from the root, stem, and leaves of *A. mongholicus*, respectively (Table 1). Then, full-length transcripts were reconstructed by using the PacBio Sequel platform. Overall, 14.21 Gb subreads were assembled, with a mean length of 1826 bp and an N50 length of 2191 bp (Additional file 1: Figure S1a). The ensuing analysis generated 643,812 reads of insert (ROIs), 305,998 of which were identified as full-length non-chimeric (FLNC) reads with a mean length of 1619 bp (Additional file 1: Figure S1b). We next applied Interactive Clustering and Error Correction (ICE) algorithm, combined with the Quiver program, for sequence clustering. After removal of redundant sequences using the CD-Hit program, 121,107 non-redundant transcripts with N50 value of 2124 bp were obtained (Table 2; Additional file 1: Figure S1c).

**Table 1:** Summary of transcriptome sequencing data from root, stem and leaves in *A. mongholicus*

Sample	Raw Reads (M)	Clean Reads (M)	Clean Bases (Gb)	Clean Reads Q20 (%)	Clean Reads Q30 (%)	Clean Reads Ratio (%)
AR-1	43.92	43.81	6.57	96.04	87.34	99.75
AR-2	44.99	44.85	6.73	96.04	87.48	99.7
AR-3	43.92	43.80	6.57	96.06	87.47	99.73
AS-1	43.92	43.81	6.57	96.15	87.63	99.75
AS-2	43.92	43.80	6.57	95.91	87.02	99.73
AS-3	45.67	45.57	6.84	96.84	88.83	99.78
AL-1	42.16	42.07	6.31	96.19	87.72	99.77
AL-2	43.92	43.82	6.57	96.10	87.50	99.77
AL-3	43.92	43.79	6.57	95.91	87.11	99.71

*AR* root, *AS* stem, *AL* leaves

**Table 2:** The non-redundant transcripts statistics

Total number	Total length	N50	N90	Max length	Min length	Sequence GC (%)
121,107	212,470,804	2124	1087	17,059	197	41.09%

### Function annotation of full-length *A. mongholicus* transcriptome

**Fig. 2:**
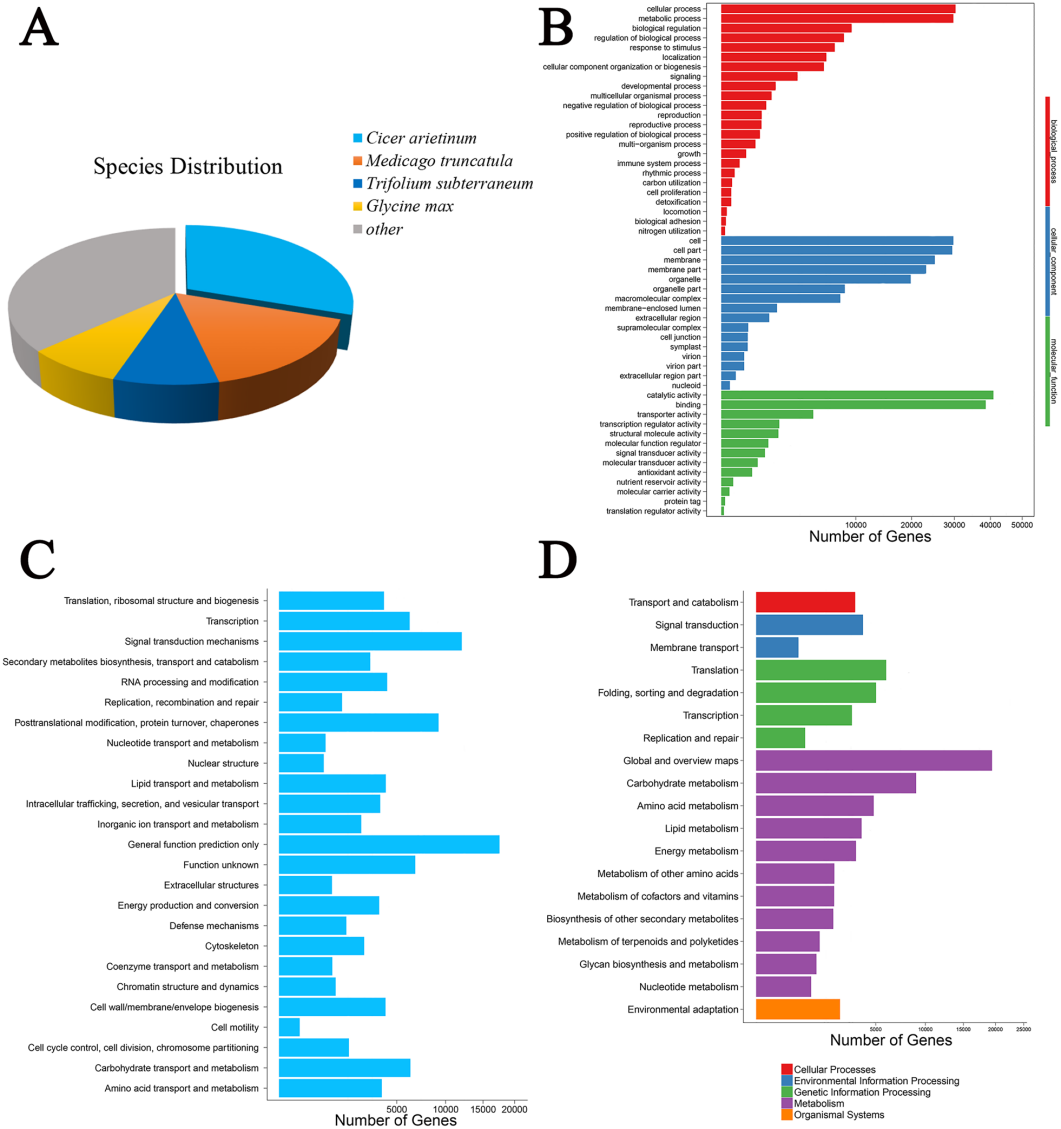
Functional annotation of transcripts of *A. mongholicus* among different samples. **a** NR homologous species distribution analysis. **b** GO classification of *A. mongholicus* transcripts. **c** KOG classification of *A. mongholicus* transcripts. **d** KEGG pathway annotation of *A. mongholicus* transcripts

To obtain a putative functional annotation of *A. mongholicus* transcriptome, 121,107 transcripts were annotated against seven protein databases: NR (non-redundant protein sequences), NT (non-redundant nucleotide sequence), SwissProt (a manually annotated and reviewed protein sequence database), KEGG (Kyoto Encyclopedia of Genes and Genomes), KOG (Clusters of Eukaryotic Orthologous Groups), Pfam (Protein families), and GO (Gene Ontology) (Table 3). After the analysis, 36,812 (30.40%) transcripts were annotated based on the information in all these databases and 104,756 (86.50%) transcripts were annotated based on information in any of these databases. Sequences of species homologous to *A. mongholicus* were analyzed by aligning the obtained sequences with those in the NR database (Fig. 2a). In this manner, 30.31, 16.13, 8.67, and 8.01% sequences were mapped to the genes of *Cicer arietinum* (Leguminosae), *Medicago truncatula* (Leguminosae), *Trifolium subterraneum* (Leguminosae), and *Glycine max* (Leguminosae), respectively.

**Table 3:** Annotation of transcripts against seven public databases

Annotated databases	Total	NR	NT	Swissprot	KEGG	KOG	Pfam	GO
Transcript Number	121,107	98,388	95,310	75,812	78,294	77,196	59,554	74,953
Percentage	100%	81.24%	78.70%	62.60%	64.65%	63.74%	49.17%	61.89%

Further, 74,953 transcripts were annotated using GO terms, in three major categories: molecular function, cellular component, and biological process (Fig. 2b; Additional file 2: Table S1). Most transcripts fell into the “cellular process” (30,306 transcripts), “cell” (29,746 transcripts), and “catalytic activity” (40,896 transcripts) categories. In addition, 77,196 transcripts were assigned to KOG and classified into 25 functional clusters (Fig. 2c; Additional file 3: Table S2). Among these, “general function prediction” (17,511 transcripts) was the most common annotation, followed by “signal transduction mechanisms” (12,029 transcripts), and “posttranslational modification, protein turnover, chaperones” (9,169 transcripts). After KEGG annotation, 78,294 transcripts were mapped to KEGG pathways in five functional categories (Cellular Processes, Environmental Information Processing, Genetic Information Processing, Metabolism, and Organismal Systems) (Fig. 2d; Additional file 4: Table S3). The “global and overview maps” (19,427 transcripts), “carbohydrate metabolism” (8934 transcripts), and “translation” (5904 transcripts) were the most representative pathways. Among them, 1156 transcripts were enriched in the “phenylpropanoid biosynthesis” pathway, 398 transcripts were annotated to “flavonoid biosynthesis”, and 170 transcripts were assigned to “isoflavonoid biosynthesis”. Importantly, 479 transcripts were annotated as related to “terpenoid backbone biosynthesis” and 173 transcripts were assigned to “sesquiterpenoid and triterpenoid biosynthesis”.

### Identification of transcription factors (TFs)

TFs play a transient, spatially regulated role in plant development and response to stress. For TF prediction, we annotated 2455 TFs belonging to 53 different TF families in the *A. mongholicus* transcriptome dataset. The C3H (219 gens), GRAS (189 gens), AP2-EREBP (178 gens), MYB (177 gens), and bHLH (176 gens) TF families contained the most members (Table 4). TFs, such as those from the MYB, bHLH, AP2-EREBP, and bZIP families, play vital roles in the regulation of isoflavonoid and terpene biosynthesis in many plant species [27–31]. After removing from consideration the TFs, whose expression levels in the three organs analyzed were extremely low [fragments per kb per million fragments (FPKM) < 1], we identified genes for 72 MYBs, 53 bHLHs, 64 AP2-EREBPs, and 11 bZIPs. Among the 72 members of the MYB family, more than half were mainly expressed in the root. For 53 bHLH genes, 18 bHLHs showed the highest expression in the root, 16 bHLHs in the stem, and 19 bHLHs in leaves. Almost all identified members of the AP2-EREBP family showed a high expression level in the root. Finally, the expression of 5 from 11 bZIP genes was the highest in the root.

**Table 4:** The number of 20 transcription factors (TFs) with different expression in different organs

TF family	Number of transcripts	Number of up-regulated transcripts		
		AR vs. AL	AR vs. AS	AL vs. AS
C3H	219	21	26	27
GRAS	189	19	38	43
AP2-EREBP	178	17	29	53
MYB	177	22	39	40
bHLH	176	48	51	34
WRKY	117	31	37	36
G2-like	90	30	28	15
NAC	85	12	27	31
ARF	73	4	12	19
ABI3VP1	69	9	5	7
C2H2	68	11	12	14
mTERF	68	18	6	3
Trihelix	65	23	14	6
C2C2-Dof	53	6	8	21
FAR1	52	6	6	3
MADS	52	20	24	16
SBP	50	6	9	11
TUB	50	2	5	5
FHA	37	14	11	0
C2C2-CO-like	36	30	23	0

*AR* root, *AS* stem, *AL* leaves

### Identification of organ-specific transcripts and differentially expressed genes (DEGs)

Overall, 58,896 transcripts were simultaneously expressed in the root, stem, and leaves (Additional file 5: Figure S2a). In addition, 6087 transcripts exhibited organ-specific expression, with 2124, 1897, and 2066 transcripts specifically expressed in the root, stem, and leaves, respectively.

**Fig. 3:**
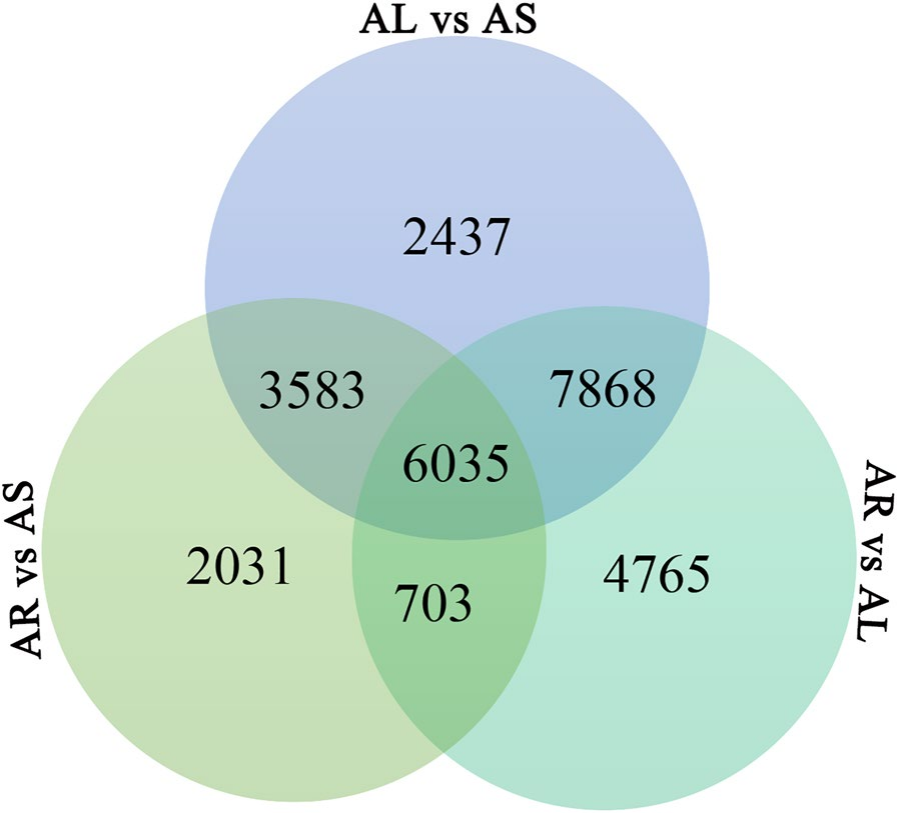
Venn diagram of DEGs in different comparisons. *AR* root, *AS* stem, *AL* leaves

DEGs in different organs (AL vs. AS, AR vs. AL, and AR vs. AS comparisons) were investigated, and the results are displayed in Fig. 3a. The AR vs. AL comparison revealed 25,970 DEGs, with 12,799 genes up-regulated and 13,171 genes down-regulated (Table 5). The highest number of specific DEGs (4765) was identified in that comparison (Fig. 3), suggesting a large biological difference between the root and leaves. For the AL vs. AS comparison, 19,923 DEGs were identified, with 12,313 genes up-regulated and 7610 genes down-regulated. Further, 18,951 DEGs were identified in the AR vs. AS comparison. Among them, 12,829 genes were up-regulated and 6122 genes were down-regulated. In all comparison groups, 6035 genes were identified as DEGs, revealing that they play an important role in the metabolism in different *A. mongholicus* organs.

**Table 5:** Number of up-regulated and down-regulated transcripts between trial groups: AR vs. AL, AR vs. AS and AL vs. AS

Compare groups	Total	Up-regulated	Down-regulated
AR vs. AL	25,970	12,799	13,171
AR vs. AS	18,951	12,829	6122
AL vs. AS	19,923	12,313	7610

*AR* root, *AS* stem, *AL* leaves

**Fig. 4:**
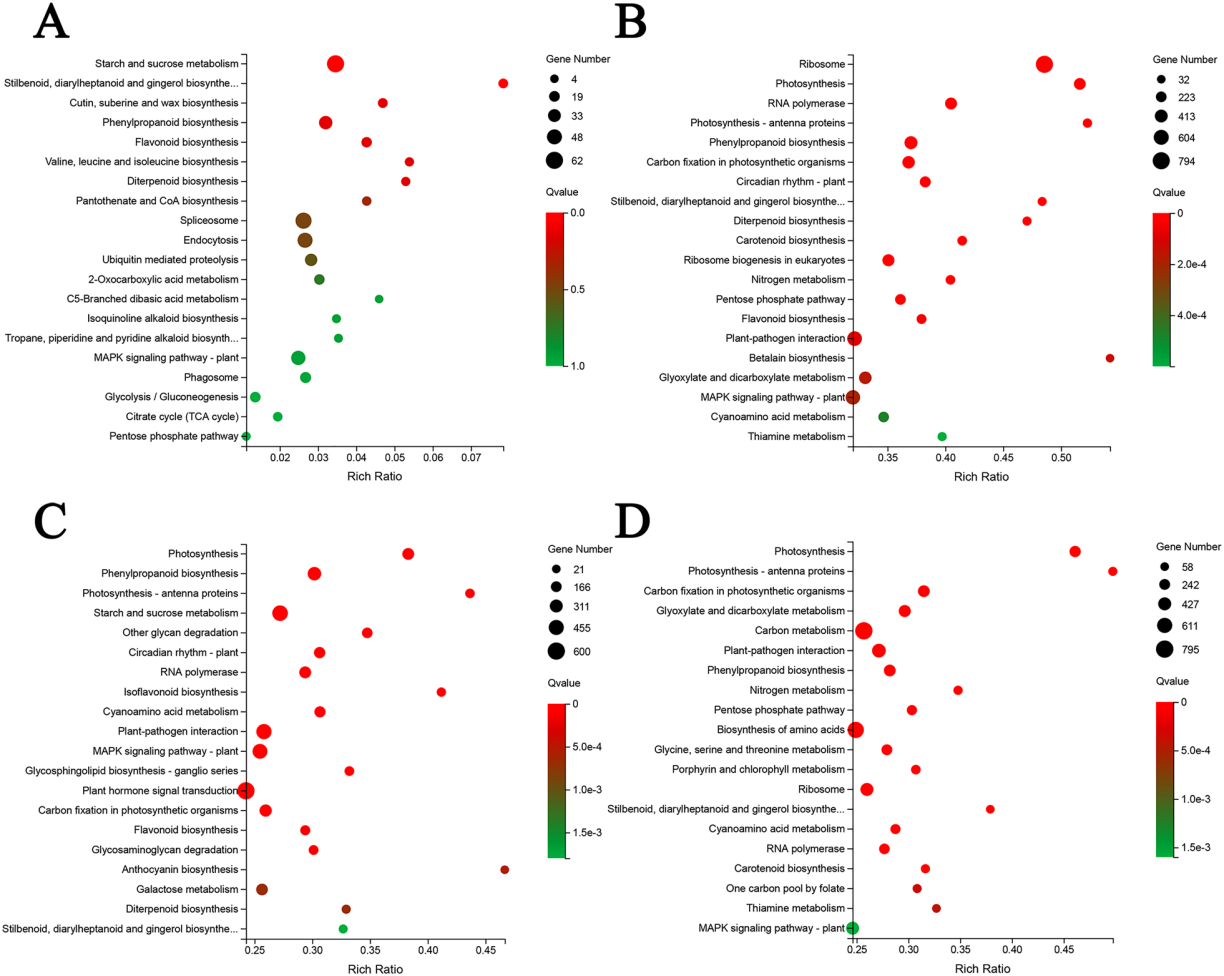
Statistic of differentially expressed genes (DEGs) in different comparisons. **a** Root-specific gens enriched in the KEGG pathway. **b** Scatterplot of the top 20 KEGG pathways enrichment of DEGs in AR vs. AL. **c** Scatterplot of the top 20 KEGG pathways enrichment of DEGs in AR vs. AS. **d** Scatterplot of the top 20 KEGG pathways enrichment of DEGs in AL vs. AS. *AR* root, *AS* stem, *AL* leaves

KEGG (Fig. 4) and GO (Additional file 5: Figure S2b–d) enrichment analyses of the DEGs from different-organ comparisons were next performed to investigate the identified transcripts in detail. In the different comparisons, the widest metabolism classes were “carbohydrate metabolism”, “amino acid metabolism”, “energy metabolism”, “lipid metabolism”, and “biosynthesis of other secondary metabolites”.

### Identification of DEGs involved in isoflavonoid biosynthesis

**Fig. 5:**
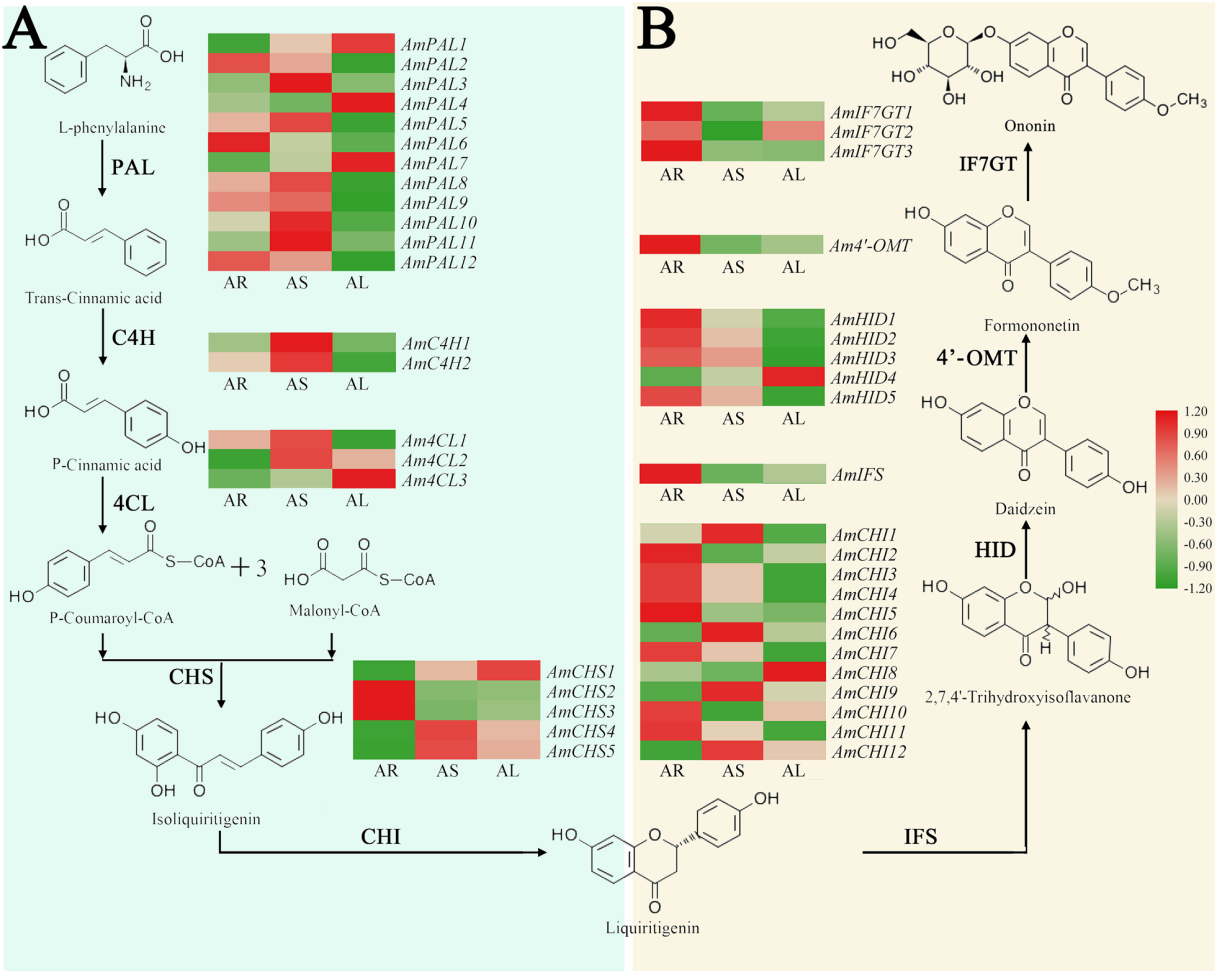
Heatmap of differentially expressed genes (DEGs) related to isoflavonoid biosynthesis. **a** Main phenylpropanoid biosynthesis and flavonoid biosynthesis of *A. mongholicus*. **b** Main isoflavonoid biosynthesis of *A. mongholicus*. *AR* root, *AS* stem, *AL* leaves

Isoflavonoids are identified almost exclusively in leguminous plants. Here, the isoflavonoid biosynthetic pathway in *A. mongholicus* was characterized based on the information for other legumes (Fig. 5) [32–35]. We identified 44 DEGs (FPKM > 1) encoding nine enzymes involved in isoflavonoid biosynthesis (Additional file 6: Table S4).

Isoflavonoids are synthesized from l-phenylalanine via a central phenylpropanoid pathway. The upstream portion of the isoflavonoid synthesis pathway is the same as that for flavonoids. For the upstream metabolism portion, 34 *A. mongholicus* DEGs were identified, including 12 *AmPALs*, two *AmC4Hs*, three *Am4CLs*, five *AmCHSs*, and 12 *AmCHIs* (Fig. 5). The expression of close to half of *AmPALs* in the root and stem was higher than that in the leaves. *AmPAL6* showed the highest expression in the root, while *AmPAL10*, *AmPAL3*, and *AmPAL11* showed the higher expression in the stem. The expression of *AmPAL1*, *AmPAL4*, and *AmPAL7* was highest in the leaves. *AmC4H1* showed the highest expression in the stem. *AmC4H2* was predominantly expressed in the stem and rarely expressed in the leaves. *Am4CL1* and *Am4CL2* showed the highest expression in the stem. *Am4CL3* showed a high expression in the leaves, unlike other *Am4CLs*. The expression of *Am4CL1* in the root was higher than that of *Am4CL2* and *Am4CL3*. *AmCHS2* and *AmCHS3* were mainly expressed in the root. Notably, the expression of *AmCHS2* and *AmCHS3* in the root was higher than that of other *CHSs*. *AmCHS4* and *AmCHS5* showed the highest expression in the stem, followed by the leaves and root. Among the 12 *AmCHIs* transcripts, more than half were most abundant in the root. *AmCHI1*, *AmCHI6*, *AmCHI9*, and *AmCHI12* were expressed predominantly in the stem. In general, genes from the phenylpropanoid and flavonoid biosynthesis pathways showed a high relative expression in the root and leaves.

Isoflavonoid biosynthetic pathway is a branch of the flavonoid pathway. The following enzymes involved in the isoflavonoid pathway in *A. mongholicus* were identified: one *AmIFSs*, five *AmHIDs*, one *Am4′-OMTs*, and three *AmIF7GTs*. The levels of candidate transcripts were investigated in detail (Fig. 5). The genes displayed differential expression in the root, stem, and leaves of *A. mongholicus*. Of note, the vast majority of candidate transcripts related to isoflavonoid biosynthesis were more highly expressed in the root than in the stems and leaves, which was consistent with the isoflavonoid content of *A. mongholicus*. The relationship between these candidate transcript levels and the determined isoflavonoid accumulation implies that these genes play major roles in the isoflavonoid biosynthesis of *A. mongholicus*.

### Analysis of DEGs in pathways related to triterpenoid saponin biosynthesis

Fifty-eight candidate genes encoding 16 enzymes related to triterpenoid saponin biosynthesis were identified by removing from consideration genes with extremely low expression (FPKM < 1). Among them, 44 were considered as DEGs (Additional file 7: Table S5) and were the focus of the ensuing detailed investigation.

**Fig. 6:**
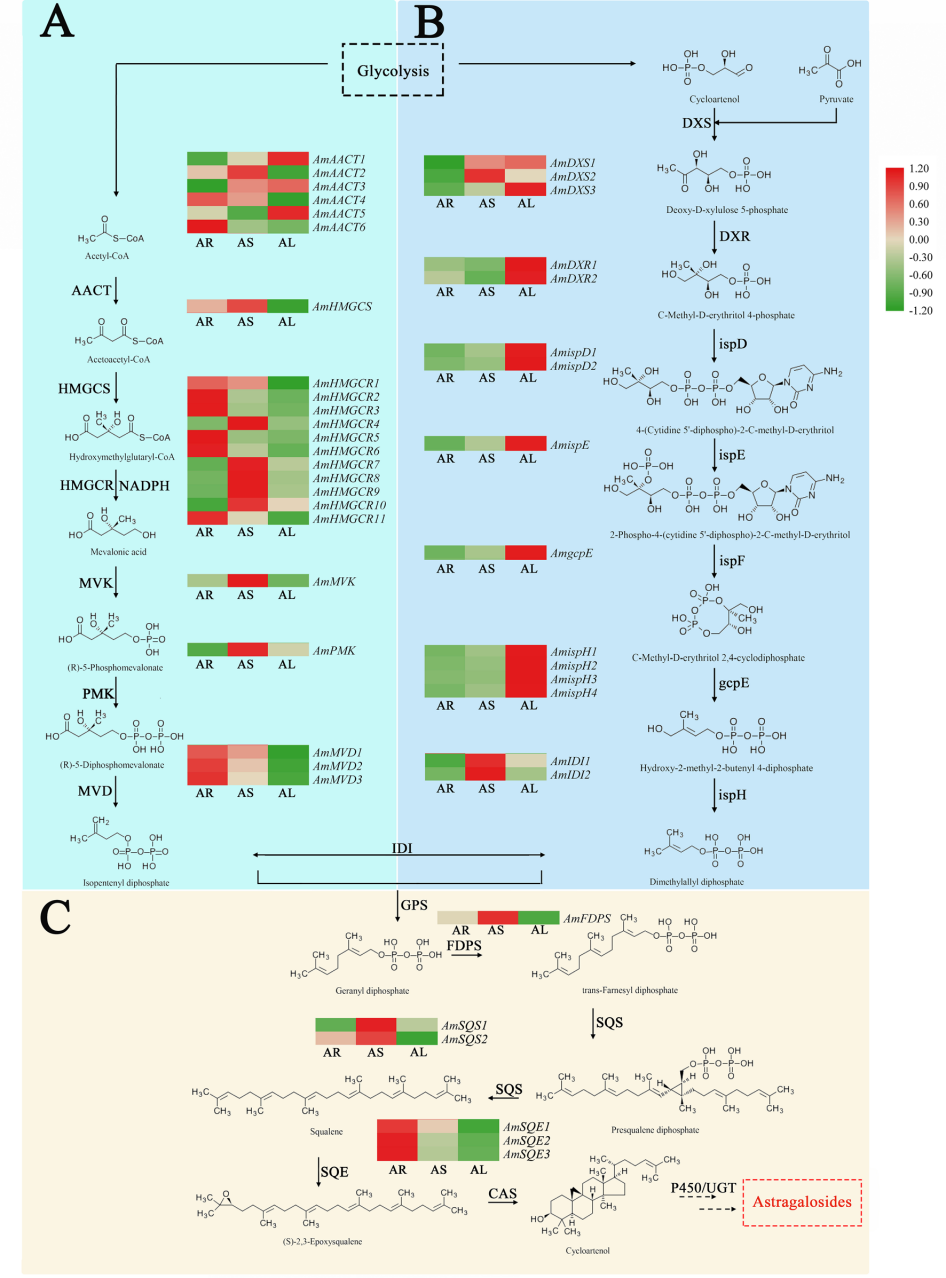
Heatmap of differentially expressed genes (DEGs) related to triterpenoid saponins biosynthesis. **a** MVA pathway. **b** MEP pathway. **c** Main sesquiterpenoid and triterpenoid biosynthesis of *A. mongholicus.* *AR* root, *AS* stem, *AL* leaves

The MVA pathway is the earliest and more traditional pathway for the synthetic biology of terpenoids. Twenty-three DEGs were identified in the MVA pathway (Fig. 6a). Half of the candidate transcripts involved in the MVA pathway were the most abundant in the root. Only a single transcript each for *AmHMGCSs*, *AmMVKs*, and *AmPMKs* was identified in the generated transcriptomic dataset as DEG involved in the MVA pathway. Further, these transcripts showed the same expression trends in the three plant organs analyzed, with the highest expression in the stem, followed by that in the root or leaves. Of the six *AmAACTs*, *AmAACT4* and *AmAACT6* showed a higher expression in the root than in the other two organs. However, the expression of *AmAACT1* and *AmAACT3* in the root was higher than that of the other *AmAACTs*, especially *AmAACT3*. These observations suggest that *AmAACT3* also plays an important role in the MVA pathway of *A. mongholicus.* HMGR is a key enzyme in the MVA pathway; accordingly, 11 *AmHMGRs* were identified as DEGs in *A. mongholicus.* Notably, the expression levels of six *AmHMGRs* in the root were higher than those in the stem and leaves. Compared with other *AmHMGRs*, *AmHMGR5* was most highly expressed in the root, which implied that the transcript plays a vital role in the MVA pathway in the *A. mongholicus* root. Three *AmMVDs* were expressed in the different organs, with the highest expression in the root, followed by that in the stem and leaves.

**Fig. 7:**
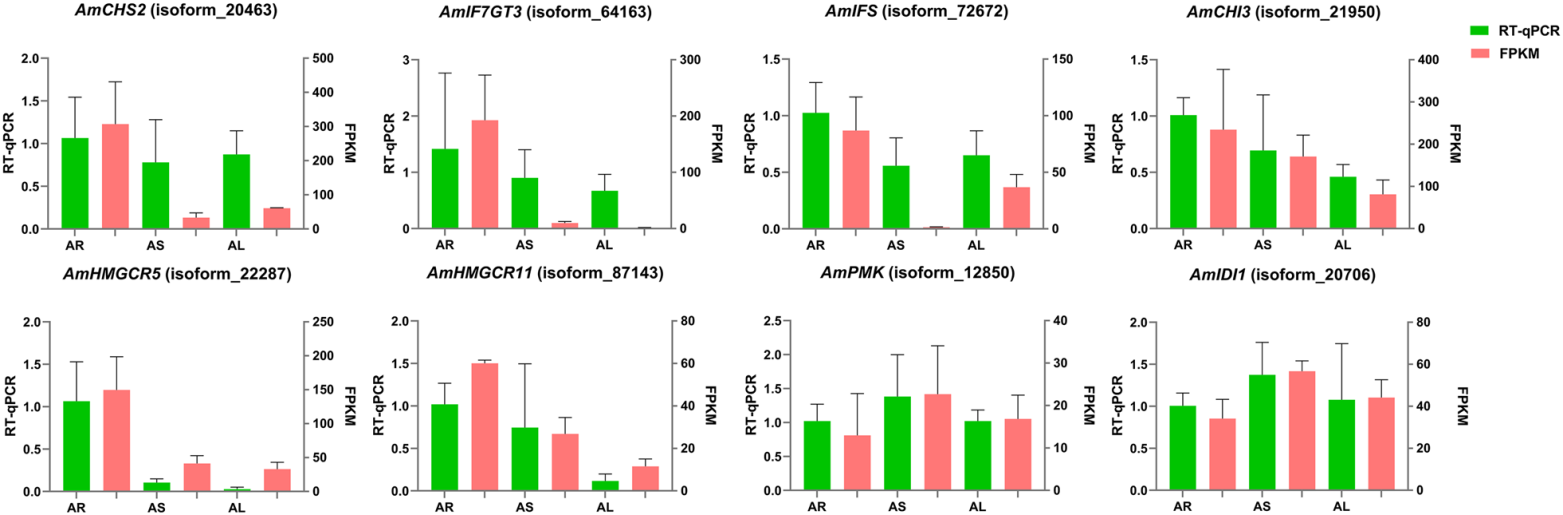
RT-qPCR validation of 8 randomly selected genes in different organs. The actin gene was *Am18S*

In the transcriptomic dataset generated in the current study, 13 DEGs were identified as involved in the MEP pathway of *A. mongholicus*, including three *AmDXSs,* two *AmDXRs*, two *AmispDs*, one *AmispE*, one *AmgcpE*, and four *AmispHs*. As shown in Fig. 6b, almost all DEGs related to the MEP pathway showed the highest expression in the leaves and lowest expression in the root. Both the MEP and MVA pathways generate IPP and its isomer DMAPP, which are precursors for terpenoid production. Triterpenoids and sesquiterpenoids are synthesized via the MVA pathway, whereas monoterpenoids, diterpenoid, and tetraterpenoids are biosynthesized via the MEP pathway. In the current study, the *A. mongholicus* MVA pathway was identified as the major pathway involved in the biosynthesis of triterpenoid saponins.

The biosynthesis of triterpenoid saponins in *A. mongholicus* mainly involves “terpenoid backbone biosynthesis” and “sesquiterpenoid and triterpenoid biosynthesis” pathways. Six DEGs encoding three enzymes were identified in the “sesquiterpenoid and triterpenoid biosynthesis” pathway (Fig. 6c). Two candidate transcripts were identified for *AmFDPSs*, but only one gene was differentially expressed. *AmFDPS* showed similar expression in the three organs analyzed, with the highest expression in the stem. *SQS* and *SQE* play a pivotal role in the biosynthetic pathway of the carbon ring skeleton of triterpenoid saponins. Three *AmSQEs* showed higher expression in the root than in the stem and leaves. The two *AmSQSs*, *AmSQS1,* and *AmSQS2*, showed the highest expression in the stems.

### Reverse transcription quantitative real-time PCR (RT-qPCR) validation of DEGs involved in flavonoid and triterpenoid saponin biosynthesis

To verify the detected gene expression levels, 10 genes were randomly selected for a real-time qPCR (RT-qPCR) experiment (Fig. 7; Additional file 8: Figure S3). The RT-qPCR data were in good agreement with the expression trends of these eight gene transcripts in the transcriptome (Fig. 7). The relative expression levels of *AmCHS2*, *AmIF7GT3*, *AmIFS, AmCHI3, AmHGCMR5* and *AmHGCMR11* were higher in root than those in stem or leaves. The relative expression of *AmPMK* and *AmIDI1* was greater in stem than that in leaves or root, which was consistent with transcription data. This result proves the accuracy of the transcriptome data.

## Discussion

Technological changes and advances in sequencing technology have a profound impact on transcriptome research of medicinal plants, enabling a quick, accurate, and efficient interpretation of gene-level information. Because of high-throughput, sensitivity, accuracy, and low cost, NGS is widely used as a research tool to reveal detailed information about the genes of medicinal plants [36–39]. Astragali Radix, a commonly used traditional Chinese medicine, is derived from the dried root of *A. mongholicus* Bunge or *A. membranaceus* (Fisch.) Bunge. Currently, transcriptome studies of *A. mongholicus* are mainly based on NGS [32, 34, 40]. Previous RNA-Seq investigations reported unigenes with an average length < 1000 bp. In another study, untargeted expressed sequence tags (EST) sequencing of *A. membranaceus* material yielded 9893 partial sequences [41]. Short-read sequencing often impacts the assembly of full-length transcripts. The reference genome of *A. mongholicus* or *A. membranaceus* has not yet been published, resulting in a lack of genetic information for this plant. SMRT provides a down-to-earth direct for the analysis of full-length transcripts in species without an assembled genome [42]. Further, SMRT has the advantages of single-molecule real-time sequencing and super-long reading length, which makes up for the shortcomings of NGS [43, 44]. Nonetheless, the high error rate of SMRT sequencing cannot be ignored. Therefore, a combination of NGS and SMRT is a widely recognized approach for the analysis of genes involved in the biosynthesis of secondary metabolites in plants [45–47]. Accordingly, we here combined NGS and SMRT sequencing to generate high-quality *A. mongholicus* transcriptome that is more complete/full-length of *A. mongholicus* transcriptome. In the current study, 643,812 CCS reads were generated, yielding 121,107 non-redundant transcript isoforms with an average length of 1619 bp and an N50 value of 2124 bp. Based on these highly accurate transcripts, 104,756 (86.50%) transcripts were successfully annotated using any of the seven databases. Of these, 16,351 transcripts might represent species-specific genes of *A. mongholicus*, whose function requires further exploration. Further, 2455 TFs representing 53 different TF families were identified. Comparisons of the gene expression in different tissues yielded 6035 shared DEGs.

Isoflavonoids, such as formononetin, ononin, calycosin, and calycosin-7-glucoside, are important bioactive compounds of *A. mongholicus*. Using UPLC-MS/MS analysis, the contents of these four isoflavonoids in the root, stem, and leaves were compared. The analysis revealed their differential accumulation in the different tissues. Nonetheless, there was a common trend of the highest isoflavonoid accumulation in the root. The tendency of active secondary compound accumulation in *A. mongholicus* is consistent with what has been reported previously [48, 49].

To reveal the relationship between isoflavonoid biosynthesis and accumulation in different plant organs, candidate genes responsible for isoflavonoid biosynthesis must be identified and the biosynthetic pathway characterized. Accordingly, KEGG pathway annotation revealed 1156 transcripts in the “phenylpropanoid biosynthesis” pathway, 398 transcripts in the “flavonoid biosynthesis” pathway, and 170 transcripts in the “isoflavonoid biosynthesis” pathway.

In the current study, we observed that transcripts from the same gene families exhibited different expression trends. The expression patterns of some key genes were consistent with isoflavonoid accumulation patterns in different organs. These included three *PALs* (*AmPAL2*, *AmPAL6*, and *AmPAL12*), two *CHSs* (*AmCHS2* and *AmCHS3*), seven *CHIs* (*AmCHI2*, *AmCHI3*, *AmCHI4*, *AmCHI5*, *AmCHI7*, *AmCHI10*, and *AmCHI11*), one *IFS* (*AmIFS*), four *HIDs* (*AmHID1*, *AmHID2*, *AmHID3*, *AmHID5*), one *4′-OMT* (*Am4′-OMT1*), and three *IF7GTs* (*AmIF7GT1*, *AmIF7GT2*, and *AmIF7GT3*). This implies that these genes play important roles in isoflavonoid biosynthesis in *A. mongholicus.* Of note, more genes upstream of the isoflavonoid synthesis pathway were expressed at high levels in the stem or leaves rather than in the root. By contrast, most genes in the middle and downstream pathways were expressed at high levels in the root. We further analyzed that the aboveground portion of *A. mongholicus* is an important biosynthesis site for phenylpropanoids and flavonoids, while the underground portion is an important biosynthesis site for isoflavonoids.

Triterpenoid saponins are important plant secondary metabolites. They have various biological activities and are widely distributed in dicotyledonous plants [50]. In the transcriptome dataset generated in the current study, 479 transcripts were annotated as related to “terpenoid backbone biosynthesis”, and 173 transcripts were assigned to “sesquiterpenoid and triterpenoid biosynthesis”. The distribution and accumulation of four triterpenoid saponins (astragaloside I, astragaloside II, astragaloside III, and astragaloside IV) in the different organs of *A. mongholicus* is shown in Fig. 1. The roots contained the highest levels of the analyzed triterpenoid saponins. Forty-four differentially expressed transcripts encoded for 16 enzymes involved in triterpenoid saponin biosynthesis. Some genes related to triterpenoid saponin biosynthesis were more highly expressed in the root than in the stem and leaves. Notably, the expression of *HMGCRs*, the key enzymes in the MVA pathway [51] (*AmHMGCR1*, *AmHMGCR2*, *AmHMGCR3*, *AmHMGCR5*, *AmHMGCR6*, and *AmHMGCR11*), was the highest in the root, in agreement with the pattern of triterpenoid saponin abundance in the different organs analyzed. Triterpenoid saponins are mainly synthesized by the MVA pathway [52]. Further, many members of the *SQS* and *SQE* families are found in plants, and some of them are key enzymes that regulate plant saponin synthesis [53]. In the current study, *AmSQE1*, *AmSQE2*, and *AmSQE3* exhibited the highest expression in the root among the three organs analyzed. Hence, the above transcripts may play important roles in triterpenoid saponin biosynthesis of *A. mongholicus*, and can potentially be used as target markers in *A. mongholicus* breeding programs aimed at increasing astragaloside production.

It has been reported that MYB, bHLH, AP2-EREBP, and bZIP families of TFs regulate isoflavonoid and triterpenoid saponin biosynthesis. We identified 72 MYB unigenes, 53 bHLH unigenes, 64 AP2-EREBP unigenes, and 11 bZIP unigenes in the current study. Interestingly, more than half of MYBs and almost all AP2-EREBPs were mainly expressed in the root, the organ with the highest accumulation of isoflavones and triterpenoid saponins in *A. mongholicus*. This suggests that MYBs and AP2-EREBPs may play an important role in the regulation of organ-specific biosynthesis of isoflavonoids and triterpenoid saponins in *A. mongholicus*. In plants, TFs mainly positively regulate secondary metabolic pathways; however, the secondary metabolic network is complex, with more branches of metabolic. Inhibition of by-product branch metabolism is used as a means to efficiently increase the metabolic flow toward specific synthetic target compounds [54, 55]. Therefore, future research should focus on *A. mongholicus* MYBs and AP2-EREBPs, to regulate the synthesis of isoflavonoids and astragalosides in that plant.

## Conclusions

In this study, the content of isoflavonoids and triterpenoid saponins in the root, stem, and leaves of *A. mongholicus* was determined. Further, *A. mongholicus* transcriptome was analyzed by a combination of short-read NGS and long-read SMRT sequencing. The analysis revealed transcripts from the two important biosynthesis pathways of *A. mongholicus* that were more accurate and complete than those reported previously. We also identified putative key transcripts and TFs involved in the isoflavonoid and triterpenoid saponin biosynthesis in this plant. Comprehensive transcriptome and metabolite analysis revealed gene expression and metabolite profiles in different plant organs, suggesting organ-specific biosynthesis, accumulation, and modification of isoflavonoid and triterpenoid saponin in *A. mongholicus*. The current study is a valuable basis for future research on gene discovery, molecular breeding, and metabolic engineering in *A. mongholicus*.

## Materials and methods

### Plant materials

Fresh roots, stems, and leaves of *A. mongholicus* (2-years old) were collected from Anhui University of Chinese Medicine (Anhui Province, China). Three independent biological replicates (i.e., derived from three plants) of each organ were washed with water, surface-dried, flash-frozen in liquid nitrogen, and then stored at – 80 °C until RNA and metabolite extraction.

### RNA extraction, quantification, and sequencing

Total RNA was extracted from fresh samples using an improved CTAB method, according to the manufacturer’s operating manual. The RNA quality was evaluated using NanoDrop and Agilent 2100 Bioanalyzer (Thermo Fisher Scientific, MA, USA). DNBSEQ platform was used for NGS sequencing. Magnetic beads with attached oligo (dT)s were used for mRNA purification. Purified mRNA was fragmented into small pieces in a fragmentation buffer, at the appropriate temperature. Then, first-strand cDNA was generated using random hexamer-primed reverse transcription, followed by second-strand cDNA synthesis. Afterward, A-tailing mix and RNA index adapters were added and the samples were incubated to allow end-repair. The obtained cDNA fragments were then PCR-amplified. The double-stranded PCR products were heat-denatured and circularized by using a splint oligo sequence to obtain the final library. Then, raw reads were filtered to obtain clean reads by removing reads containing adaptors, poly-N stretched, or low quality reads.

For SMRT sequencing, the first-strand cDNA was synthesized from 800–1000 ng total RNA by using SMARTer PCR cDNA Synthesis Kit (Clontech, Japan). Second-strand cDNA synthesis was performed using SMARTScribe™ Reverse Transcriptase (Clontech). Double-stranded cDNA was produced by large-scale PCR using PrimeSTAR GXL DNA Polymerase (Clontech). Double-stranded cDNA fragments 0–5 kb in length were selected. The sequencing was done using PacBio Sequel sequencer. Library preparation and sequencing were done at BGI (Shenzhen, Guangdong Province, China).

### Analysis of Iso-Seq data

Raw sequencing data generated by the Pacific Bioscience Sequel sequencer were processed using the SMRT analysis package version 2.3.0 (Pacific Biosciences, https://www.pacb.com/products-and-services/analytical-software/smrtanalysis). Raw polymerase reads with full pass > 0 and predicted consensus accuracy > 0.75 were selected to produce ROI. ROIs with a minimum length of 300 bp were classified into full-length non-chimeric and non-full-length transcript sequences based on whether the 5′-primer, 3′-primer, and poly-A tail were detected. The full-length sequences were processed to consensus isoforms by using the ICE algorithm and then polished using Quiver quality-aware algorithm. To improve the accuracy of full-length transcript calling, the high error rates of SMRT subreads were corrected using NGS reads by using Long-Read De Bruijn Graph Error Correction (LoRDEC) [56]. High-quality consensus isoforms (expected Quiver accuracy ≥ 0.95) from each library were merged and redundant isoforms were removed using CD-HIT [57] (parameters: -c 0.98 -T 6 -G 0 -aL 0.90 -AL 100 -aS 0.98 -AS 30) based on sequence similarity to obtain final unique full-length isoforms.

Final full-length isoforms were mapped to the NR, NT, SwissProt, KEGG (version 59), KOG, and Pfam databases by using Blast software (version 2.2.23) [58] with default parameters (E-value threshold ≤ 10^–5^) to obtain isoform annotations. GO annotations and functional classifications were obtained using Blast2GO program (version 2.5.0, E-value ≤ 10^–5^) [59] based on NR annotations. TF were predicted by mapping to PlntfDB.

### DEG analysis

Clean reads from all samples were mapped to the full-length transcriptome using Bowtie2 (version 2.2.5) [60]. The expression of unigenes was then calculated by using RSEM (version 1.2.12) [61]. FPKM values were used to quantify the expression of each unigene. DEGs were identified using DEseq2 [62], at Q-value (adjust P-value) < 0.001 and fold change ≥ 2 or ≤ –2. The identified DEGs were annotated using GO terms and KEGG database with Phyper in the R package, at Q-value ≤ 0.05 [63]. The DEG FPKM values were greater than or equal to 1.

### Reverse transcription quantitative real-time PCR (RT‑qPCR) analysis

The expression of 10 randomly selected genes from the transcriptome database was validated by RT-qPCR. The expression of the actin gene was used a normalization control. Primer sequences are listed in Additional file 9: Table S6. Total RNA from the root, stem, and leaves of *A. mongholicus* was reverse-transcribed into the first-strand cDNA using PrimeScript II 1st Strand cDNA Synthesis Kit (Takara, Japan). RT-qPCR analysis of gene expression was performed using three biological replicates and three technical replicates, with TB Green® Premix Ex Taq™ II (Takara) and Agilent StrataGene Mx3000P qPCR System (Agilent, USA). The relative gene expression was analyzed using the 2^−ΔΔCT^ method.

### Determination of isoflavonoid and triterpenoid saponin content

Dried and homogenized powder (0.2 g) obtained from the root, stem, or leaves of *A. mongholicus* was accurately weighted and suspended in 4 mL of 75% methanol. The samples were ultrasonicated at room temperature for 60 min and the supernatant collected. The solution was filtered through a 0.22 μm membrane filter, and then injected 1μL into the UPLC-MS/MS system for analysis. Each sample was analyzed in triplicate. Chromatographic separation was performed using an ExionLC AD (SCIEX, USA), an Acquity UPLC BEH C18 column (100 mm × 2.1 mm × 1.7 mm), and a C18 Pre-column (2.1 mm × 100 mm × 1.7 μm; Waters). Gradient elution (flow rate of 0.2 mL/min) with acetonitrile as solvent A and water with 0.1% formic acid as solvent B was used. The elution gradient was set as follows: 0–2 min (8–40% A), 2–5 min (40–55% B), 5–7 min (55–60% B), 7–8 min (60–80% B), and 8–10 min (80–8% B). The column temperature was maintained at 28 °C, and the column was equilibrated for 2 min between individual runs.

An AB SCIEX Triple Quad 4500 (AB SCIEX, USA) equipped with an electrospray ionization source was used to detect the MS signals. The MS detection was performed in positive ion and multiple reaction monitoring (MRM) modes, with the following parameters: capillary voltage operated at 5.5 kV; source temperature of 550 °C; nebulizer gas at 55 psi; and curtain gas at 35 psi. The cone voltage (CV) and collision energy (CE) were set to match the MRM of each marker (Additional file 10: Table S7). Data were acquired with MultiQuant software.

## Supplementary Information

**Additional file 1**: **Figure S1**. Sequencing results of Pacbio sequel platform. a Length distribution of subreads. b The number and length distributions of FLNC reads. c The number and length distributions of non-redundant transcript isoforms.

**Additional file 2**: **Table S1**. GO annotation of the *A. mongholicus* transcripts.

**Additional file 3**: **Table S2**. KOG annotation of the *A. mongholicus* transcripts.

**Additional file 4**: **Table S3**. KEGG pathway annotation of *A. mongholicus*.

**Additional file 5**: **Figure S2**. Statistics of transcripts and differentially expressed genes. a Venn diagram of transcripts in different organs. b, c, d Scatterplot of the GO functional enrichment of DEGs (AR vs. AL, AR vs. AS and AL vs. AS, respectively).

**Additional file 6**: **Table S4**. Differentially expressed genes involved in isoflavonoid biosynthesis.

**Additional file 7**: **Table S5**. Differentially expressed genes involved in triterpenoid saponins biosynthesis.

**Additional file 8**: **Figure S3**. RT-qPCR validation of *AmAACT3*, *AmDXS1* in different organs. The actin *gene* was *Am18S*.

**Additional file 9**: **Table S6**. Primers used in validation experiment of gene expression by RT-qPCR. (AR vs. AL, AR vs. AS and AL vs. AS, respectively).

**Additional file 10**: **Table S7**. Optimized mass spectra conditions.

## Data Availability

The sequencing data of all sample-sequencing results have been submitted to the NCBI SRA (PRJNA681346) (https://dataview.ncbi.nlm.nih.gov/object/PRJNA681346?reviewer=cf9fmfe1blgqlaej16ksehqjrt).

## Abbreviations

CCS: Circular consensus sequencing
NR: Non-redundant protein
NT: Non-redundant nucleotide sequence
GO: Gene Ontology
KEGG: Kyoto Encyclopedia of Genes and Genomes
KOG: Clusters of Eukaryotic Orthologous Groups
Pfam: Protein family
PAL: Phenylalanine ammonialyase
C4H: Trans-cinnamate 4-monooxygenase
4CL: 4-Coumarate CoA ligase
CHS: Chalcone synthase
CHI: Chalcone isomerase
IFS: 2-Hydroxyisoflavanone synthase
HID: 2-Hydroxyisoflavanone dehydratase
4’-OMT: 2,7,4'-Trihydroxyisoflavanone
IF7GT: Isoflavone 7-O-glucosyltransferase
I3’H: Isoflavone 3'-hydroxylase
I2’H: Isoflavone 2'-hydroxylase
AACT: Acetyl-CoA C-acetyltransferase
HMGCS: Hydroxymethylglutaryl-CoA synthase
HMGCR: Hydroxymethylglutaryl-CoA reductase
MVK: Mevalonate kinase
PMK: Phosphomevalonate kinase
MVD: Diphosphomevalonate decarboxylase
DXS: 1-deoxy-D-xylulose-5-phosphate synthase
DXR: 1-deoxy-D-xylulose-5-phosphate reductoisomerase
ispD: 2-C-methyl-D-erythritol 4-phosphate cytidylyltransferase
ispE: 4-diphosphocytidyl-2-C-methyl-D-erythritol kinase
ispF: 2-C-methyl-D-erythritol 2,4-cyclodiphosphate synthase
gcpE: (E)-4-hydroxy-3-methylbut-2-enyl-diphosphate synthase
ispH: 4-hydroxy-3-methylbut-2-en-1-yl diphosphate reductase
IDI: Isopentenyl-diphosphate Delta-isomerase
GPS: Geranylgeranyl diphosphate synthase
FDPS: Farnesyl diphosphate synthase
SQS: Squalene synthase
SQE: Squalene epoxidase
CAS: Cycloartenol synthase
UGT: UDP-glycosyltransferase
