# Supplementary material for: Full-length transcriptome sequences by a combination of sequencing platforms applied to isoflavonoid and triterpenoid saponin biosynthesis of Astragalus mongholicus Bunge
Source: Plant Methods. 2021 Jun 15;17:61. doi: 10.1186/s13007-021-00762-1 (PMC8207730; doi:10.1186/s13007-021-00762-1)
Supplement: Supplementary file 1 — Additional file 1: Figure S1. Sequencing results of Pacbio sequel platform. a Length distribution of subreads. b The number and length distributions of FLNC reads. c The number and length distributions of non-redundant transcript isoforms. [file 13007_2021_762_MOESM1_ESM.doc]

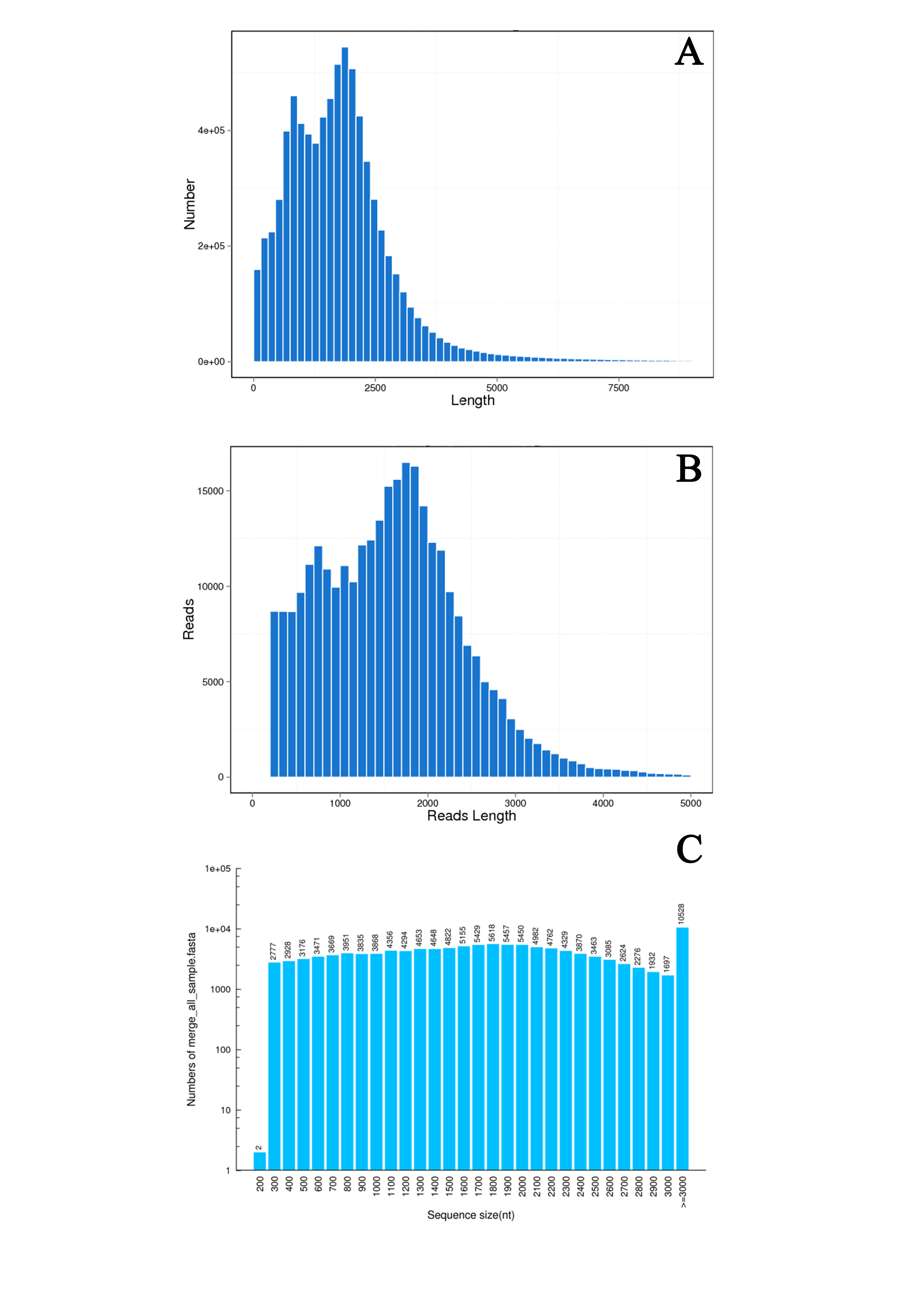


Figure S1. Sequencing results of PacBio sequel platform. a Length distribution of subreads. b The number and length distributions of FLNC reads. c The number and length distributions of non-redundant transcript isoforms.
