# Supplementary material for: Full-length transcriptome sequences by a combination of sequencing platforms applied to isoflavonoid and triterpenoid saponin biosynthesis of Astragalus mongholicus Bunge
Source: Plant Methods. 2021 Jun 15;17:61. doi: 10.1186/s13007-021-00762-1 (PMC8207730; doi:10.1186/s13007-021-00762-1)
Supplement: Supplementary file 5 — Additional file 5: Figure S2. Statistics of transcripts and differentially expressed genes. a Venn diagram of transcripts in different organs. b, c, d Scatterplot of the GO functional enrichment of DEGs (AR vs. AL, AR vs. AS and AL vs. AS, respectively). [file 13007_2021_762_MOESM5_ESM.doc]

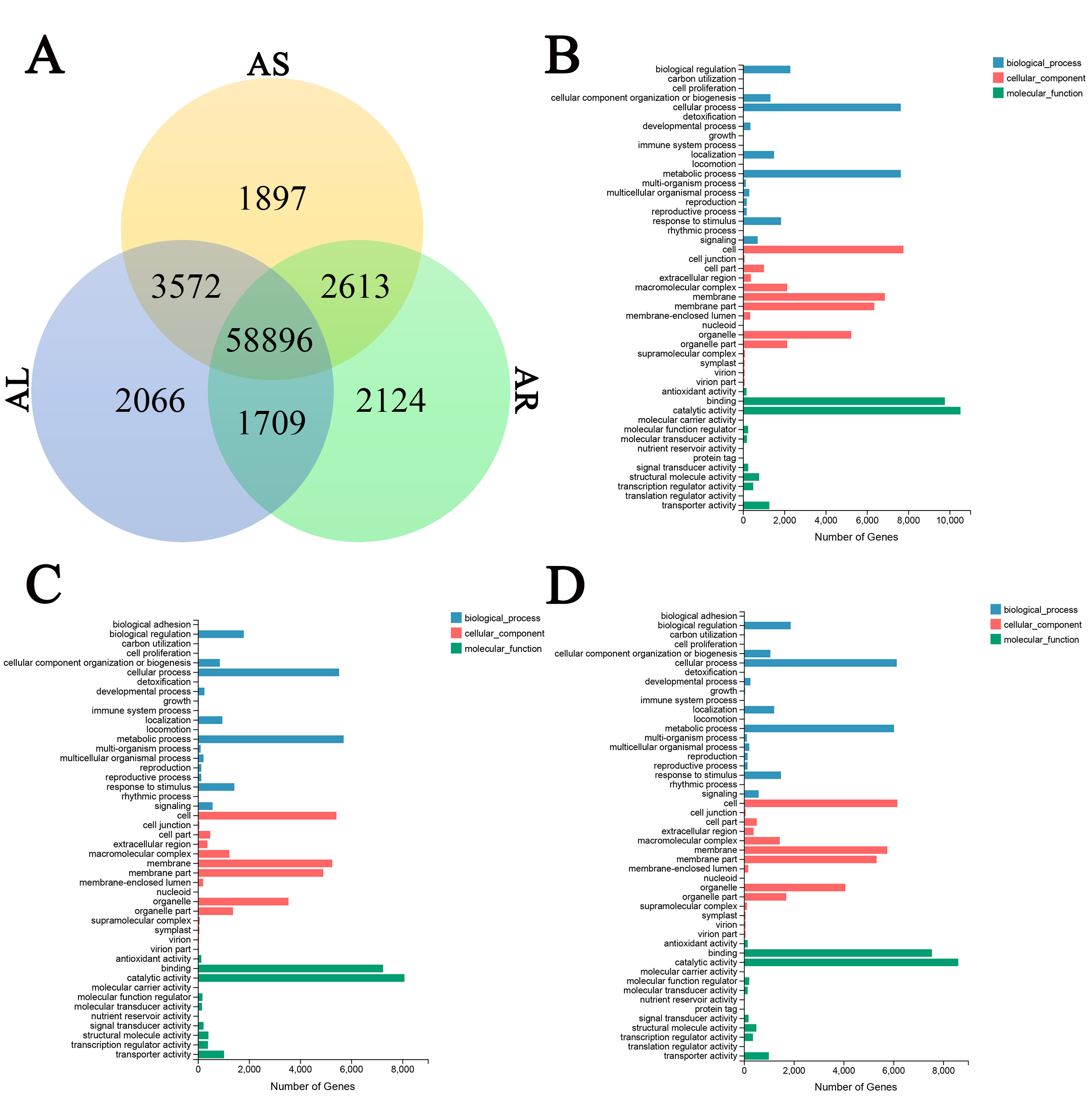


Figure S2. Statistics of transcripts and differentially expressed genes. **a** Venn diagram of transcripts in different organs. **b, c, d** Scatterplot of the GO functional enrichment of DEGs (AR vs. AL, AR vs. AS and AL vs. AS , respectively). AR, Roots; AS, Stems; AL, Leaves.
