# Supplementary figures and images for: Full-length transcriptome sequences by a combination of sequencing platforms applied to isoflavonoid and triterpenoid saponin biosynthesis of Astragalus mongholicus Bunge
Source: Plant Methods. 2021 Jun 15;17:61. doi: 10.1186/s13007-021-00762-1 (PMC8207730; doi:10.1186/s13007-021-00762-1)

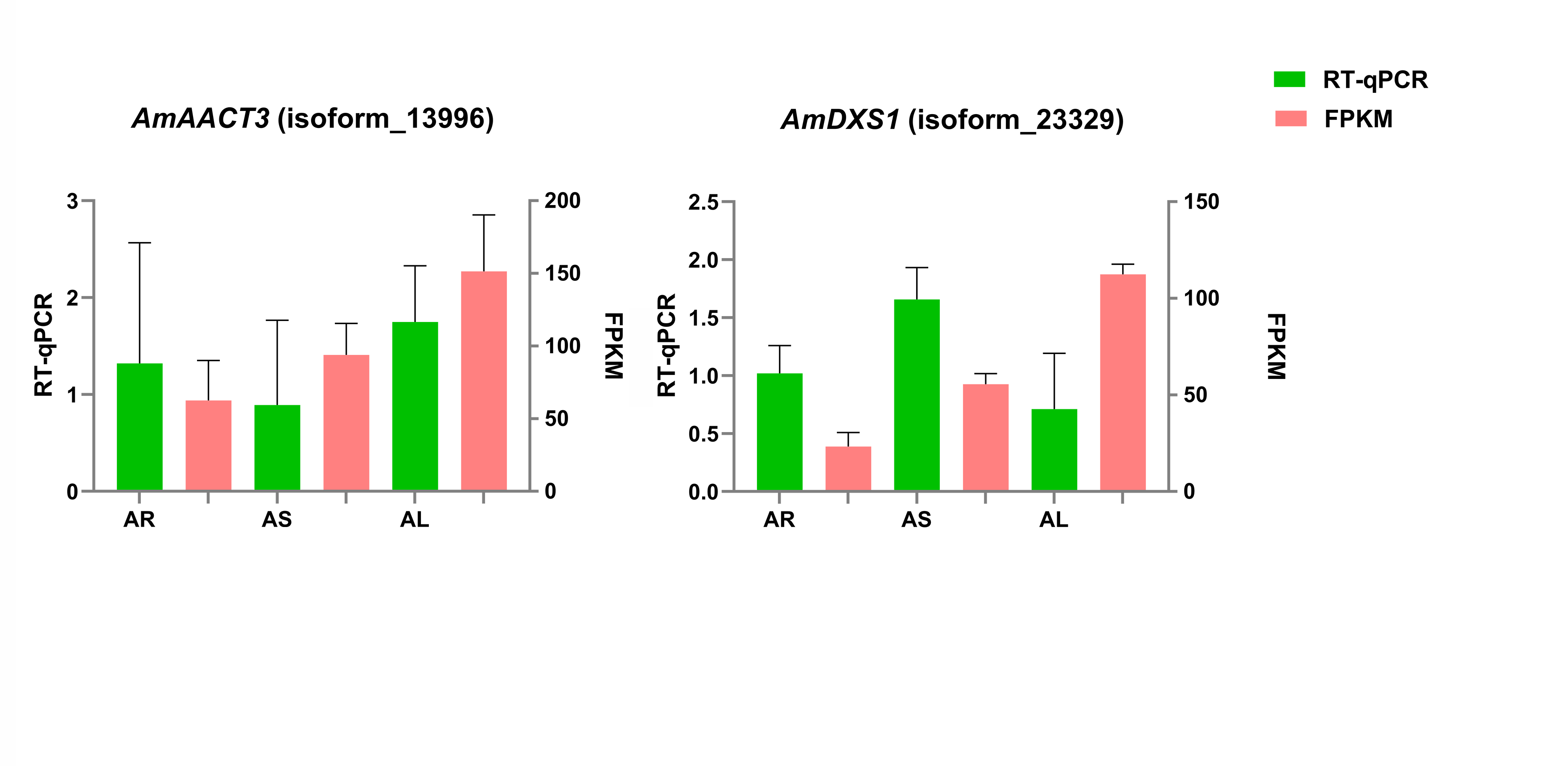


Figure S3. RT-qPCR validation of *AmAACT3*, *AmDXS1* in different organs. The *actin* gene was *Am18S.*

Supplement: Supplementary file 8 — Additional file 8: Figure S3. RT-qPCR validation of AmAACT3, AmDXS1 in different organs. The actin gene was Am18S. [file 13007_2021_762_MOESM8_ESM.doc]
